# Supplementary material for: Dissecting extracellular and intracellular distribution of nanoparticles and their contribution to therapeutic response by monochromatic ratiometric imaging
Source: Nat Commun. 2022 Apr 14;13:2004. doi: 10.1038/s41467-022-29679-6 (PMC9010411; doi:10.1038/s41467-022-29679-6)
Supplement: Supplementary file 1 — Supplementary Information [file 41467_2022_29679_MOESM1_ESM.pdf]

Supplementary Information

Dissecting extracellular and intracellular distribution of nanoparticles and their contribution to therapeutic response by monochromatic ratiometric imaging

Yue Yan[1,2]*, Binlong Chen[1,2]*, Qingqing Yin[2], Zenghui Wang[2], Ye Yang[2], Fangjie Wan[2], Yaoqi Wang[2], Mingmei Tang[2], Heming Xia[2], Meifang Chen[2], Jianxiong Liu[2], Siling Wang[3], Qiang Zhang[1,2], Yiguang Wang[1,2]†

[1]State Key Laboratory of Natural and Biomimetic Drugs, Peking University, Beijing 100191, China.

[2]Beijing Key Laboratory of Molecular Pharmaceutics and New Drug Delivery Systems, School of Pharmaceutical Sciences, Peking University, Beijing 100191, China.

[3]School of Pharmacy, Shenyang Pharmaceutical University, Shenyang, Liaoning 110016, China.

†Corresponding author. Email: yiguang.wang@pku.edu.cn

*These authors contributed equally to this work.

This PDF file includes:

Supplementary Figures 1-19 and Supplementary Table 1

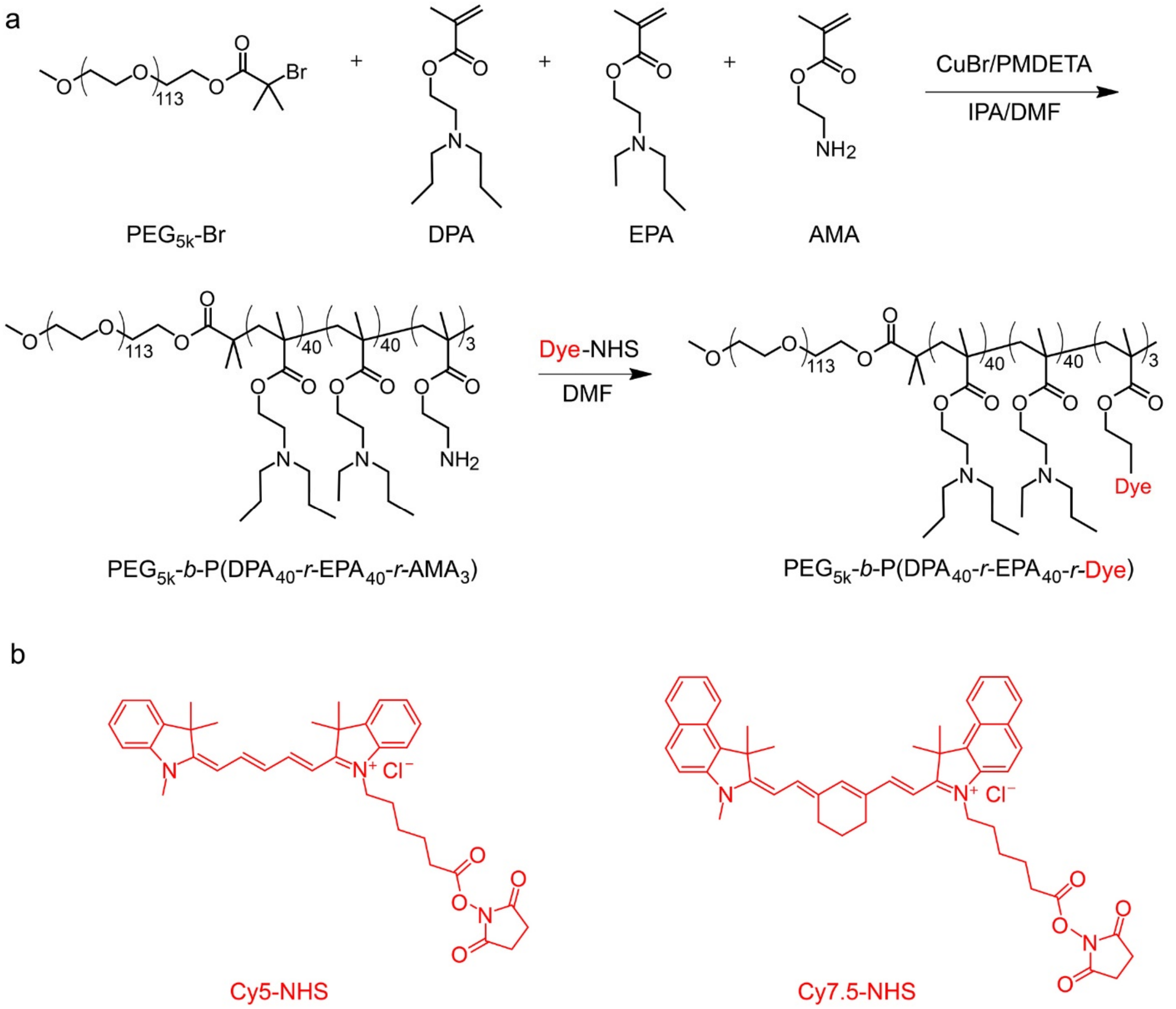

Supplementary Figure 1. Syntheses of dye-conjugated functional polymers. (a) Synthetic routes of PEG_{5k}-*b*-P(DPA_{40}-*r*-EPA_{40}-*r*-AMA_3) and dye-conjugated functional polymers. (b) The chemical structures of Cyanine5 NHS ester (Cy5-NHS) and Cyanine7.5 NHS ester (Cy7.5-NHS).

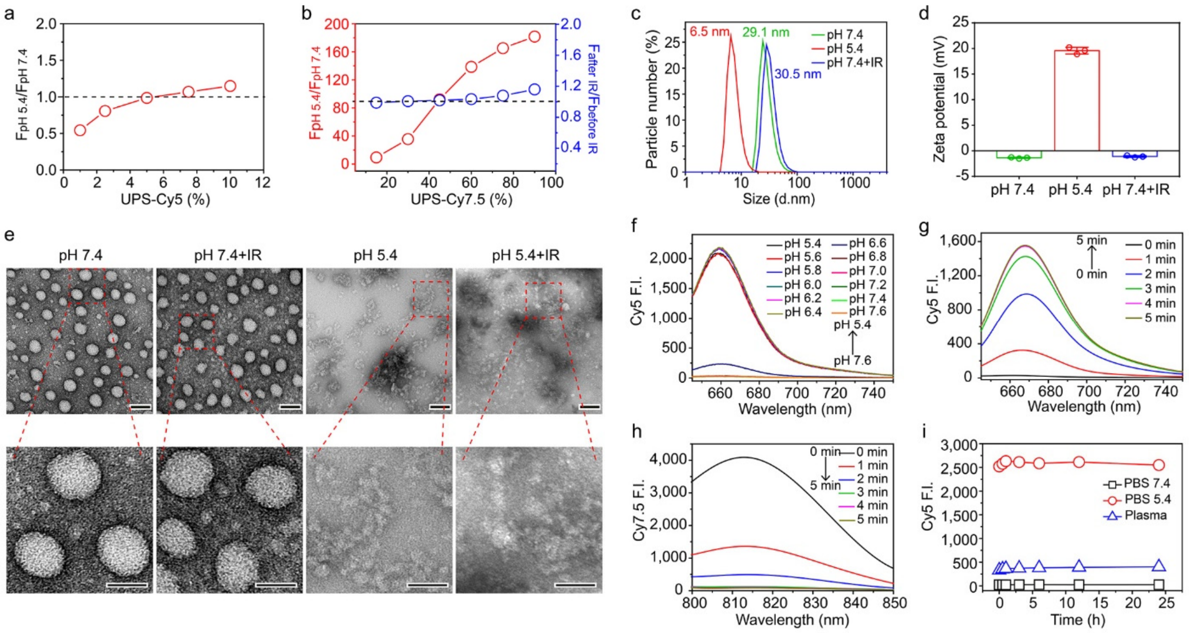

Supplementary Figure 2. Characterization of MRIN. (a) The development of Cy5 signal always on micelle. Cy5 fluorescence intensity ratios ($F_{pH\ 5.4}/F_{pH\ 7.4}$) as a function of the molar ratio of UPS-Cy5 polymer over dye-free UPS polymer in the hybrid micelles. (b) Cy5 fluorescence intensity ratios ($F_{pH\ 5.4}/F_{pH\ 7.4}$ and $F_{after\ IR}/F_{before\ IR}$) as a function of molar ratio of UPS-Cy7.5 in MRIN. (c) The particle size distribution of MRIN at pH 5.4 and pH 7.4 with or without 808 nm laser irradiation. (d) Zeta potentials of MRIN at pH 5.4 and pH 7.4 with or without irradiation. Data were presented as mean ± s.d. (n = 3 biologically independent experiments). (e) The TEM images of MRIN with high resolution. Scale bar: 50 nm for the top panel, and 25 nm for the bottom panel. (f) pH-dependent fluorescence spectra of MRIN. Each sample was excited at 630 nm, and the corresponding emission spectrum was collected from 645 to 750 nm. (g-h) NIR switched fluorescence recovery of MRIN. Fluorescence spectra changes of Cy5 (g) and Cy7.5 (h) upon 808 nm irradiation (0.5 W cm^{-2}) for different time. (i) The stability of MRIN in PBS 7.4, PBS 5.4 buffers and plasma, respectively (Cy5 concentration of 0.25 μg mL^{-1}).

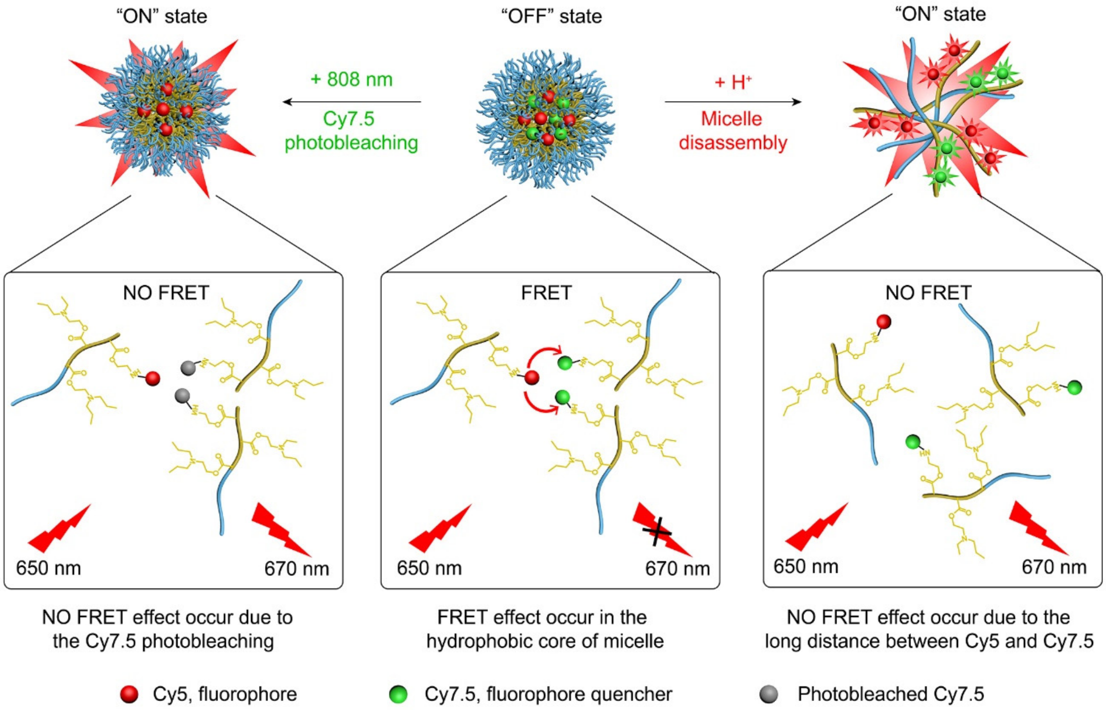

Supplementary Figure 3. The 'turn-on' mechanism of MRIN based on pH-induced dissociation and 808 nm laser irradiation. In the MRIN platform, Cy7.5 served as a fluorescence quencher of Cy5 through FRET effect. Tertiary amino groups are incorporated into polymers as ionizable groups to impart pH sensitivity. When pH > 6.3, the hydrophobic segments of PEG_{5k}-*b*-P(DPA_{40}-*r*-EPA_{40}-*r*-Dye) polymer self-assemble into the micelle cores, leading to fluorescence quenching of Cy5 fluorophore by hetero-FRET. In the micelle state, the Cy5 fluorescence signal can be recovered by photobleaching Cy7.5 with 808 nm irradiation. When pH < 6.3, protonation of the PEG_{5k}-*b*-P(DPA_{40}-*r*-EPA_{40}-*r*-Dye) segments results in micelle dissociation, leading to abolishment of FRET effect due to the long distance between fluorophore and fluorophore quencher.

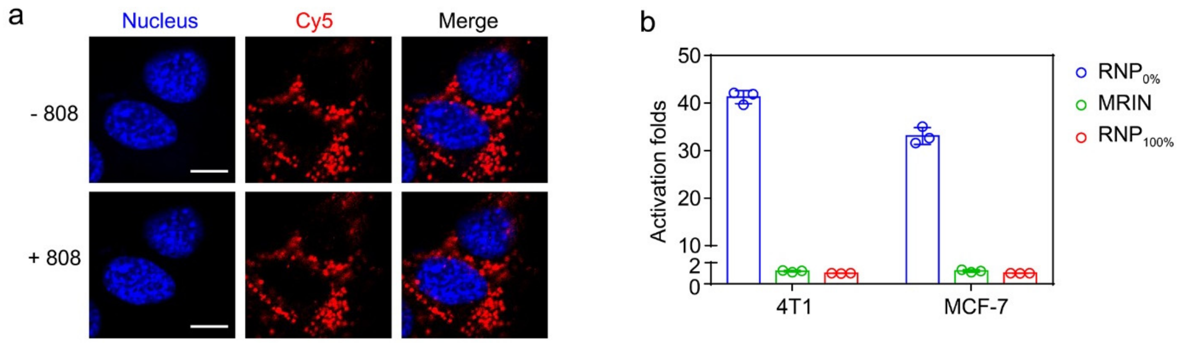

Supplementary Figure 4. The intracellular fluorescence behavior of three nanoparticles. (a) The intracellular Cy5 fluorescence images of MRIN in 4T1 cells before and after 808 nm irradiation. Scale bar = 10 μm. (b) The intracellular fluorescence intensity of three nanoparticles in 4T1 cell suspension before and after 808 nm irradiation measured by flow cytometry. Data were presented as mean ± s.d. (n = 3 biologically independent cell samples).

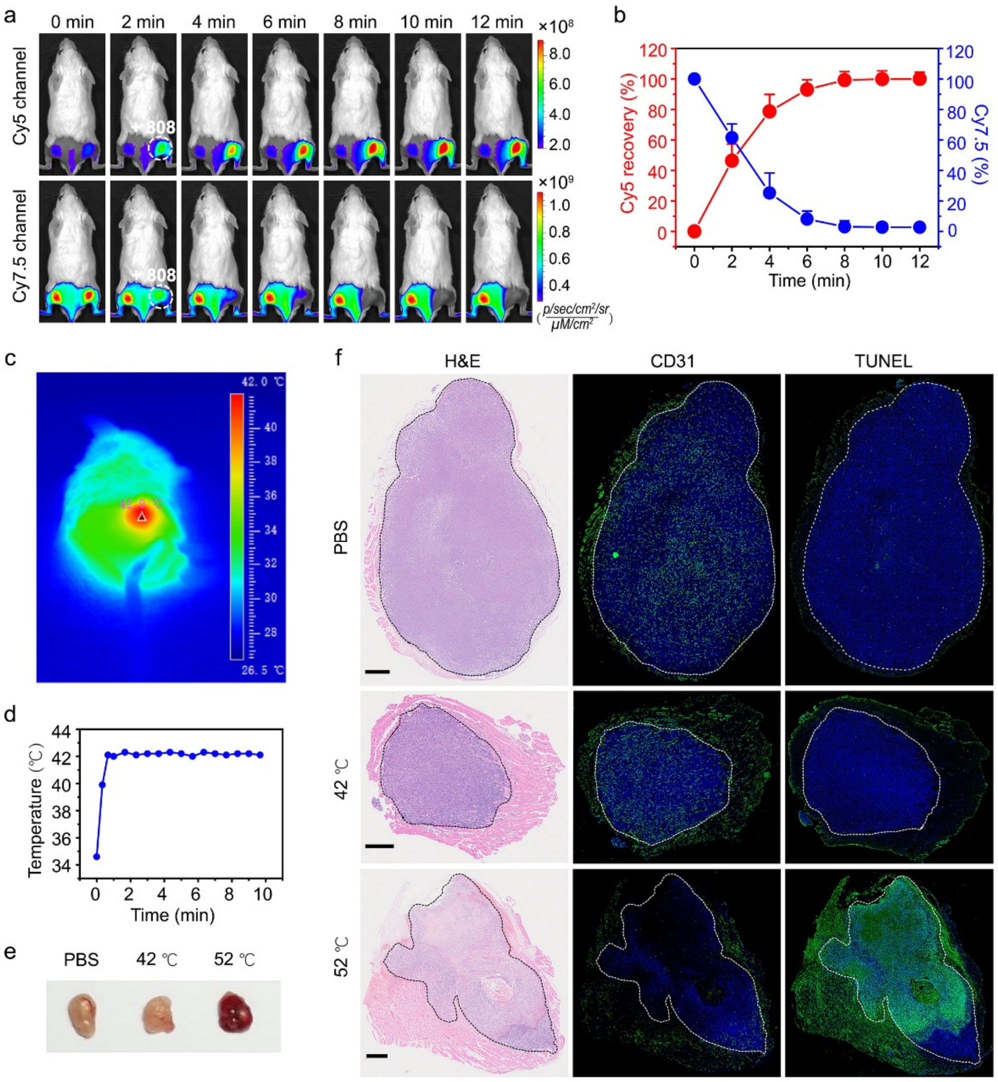

Supplementary Figure 5. *In vivo* irradiation conditions selection and safety. (a) *In vivo* fluorescence images of the bilateral 4T1 tumour-bearing mice with different irradiation time at 24 h post-injection of MRIN (75 μg kg^{-1} Cy5). (The circles indicate the irradiation sites of right tumours. During irradiation, the real-time temperature of the tumour was kept at about 42 °C). (b) The corresponding percentages of Cy5 fluorescence recovery and Cy7.5 fluorescence residual of 4T1 tumours upon different irradiation times, quantified from the results of Fig. S5a. Data were presented as mean ± s.d. (n = 5 biologically independent mice). (c) *In vivo* IR thermal imaging of mouse under irradiation. (d) During 808 nm irradiation, the real-time temperature of the tumour was monitored by an infrared thermal camera to keep it at about 42 °C by changing the laser power. (e) The photographic images of excised tumours after laser irradiation at different temperature. (f) H&E staining, tumour vessels labeled with anti-CD31 antibody and TUNEL staining images of tumour slices for different treatment groups. (The white dotted lines indicate the tumour sites). Scale bar = 800 μm.

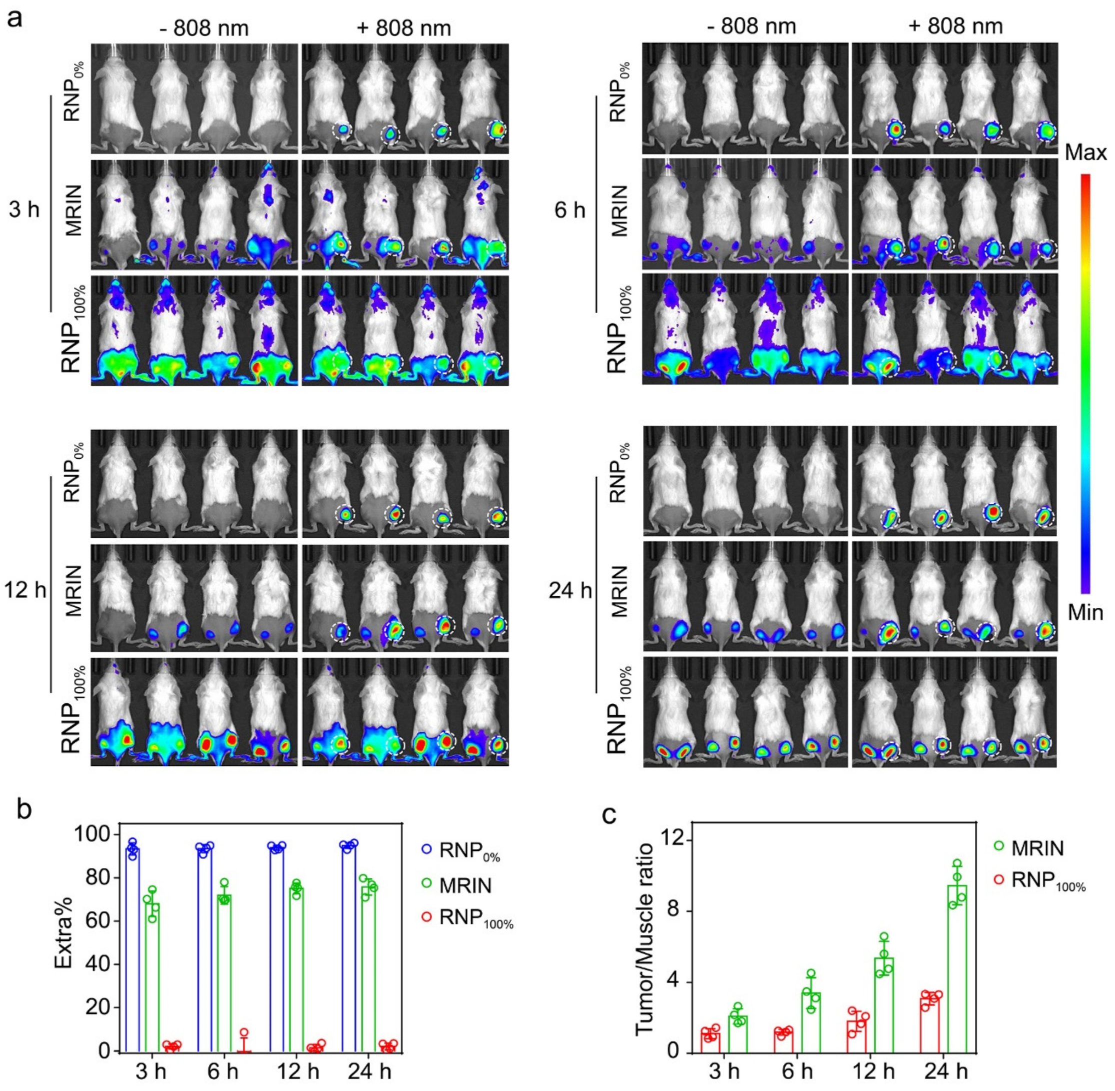

Supplementary Figure 6. *In vivo* fluorescence images of mice after treatment with different micelles. (a) *In vivo* fluorescence images of the bilateral 4T1 tumour-bearing mice with or without 808 nm irradiation on right tumours at 3, 6, 12, and 24 h post-injection of different micelles (equivalent Cy5 concentration of 75 μg kg^{-1}). (The circles indicate the irradiation sites of right tumours. During irradiation, the real-time temperature of the tumour was kept at about 42 °C). (b) The quantified extracellular percentages of different micelles *in vivo* at 3, 6, 12, 24 h post-injection from the results of Fig. S6a (n = 4 biologically independent mice). (c) The quantified Cy5 fluorescence ratio of tumour to adjacent normal tissues from the results of Fig. S6a (n = 4 biologically independent mice). All data were presented as mean ± s.d..

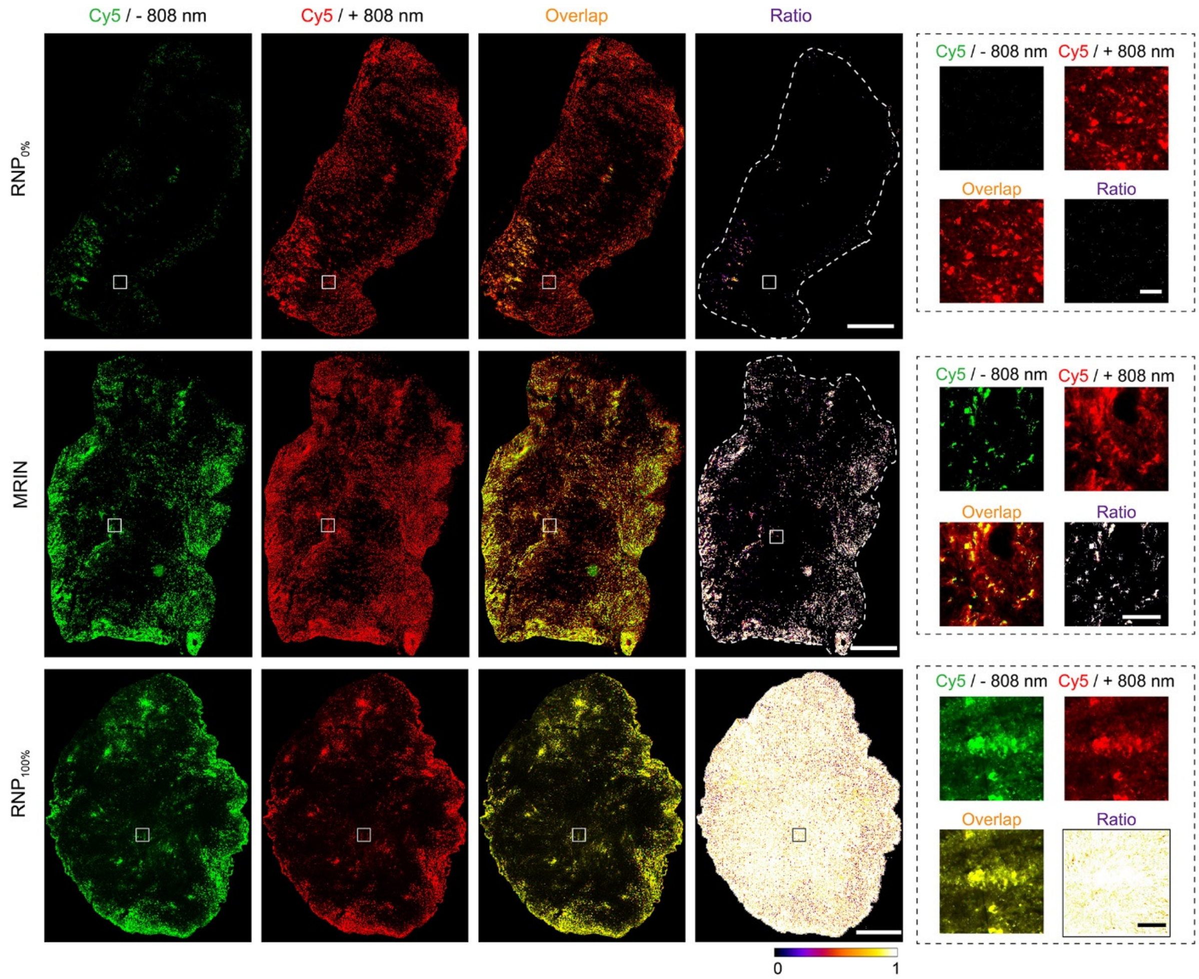

Supplementary Figure 7. The fluorescence images of the tumour slides from mice treated with different micelles. Left panel: Whole tumour slide scanning. The pre-irradiated and post-irradiated Cy5 signals were presented in green and red, respectively. The overlap and ratio channels were generated from the overlap and ratio of post-irradiated Cy5 fluorescence image and the pre-irradiated Cy5 fluorescence image, respectively. Scale bar = 800 μm. Right panel: The zoom-in images of squares region in left panel. Scale bar = 50 μm.

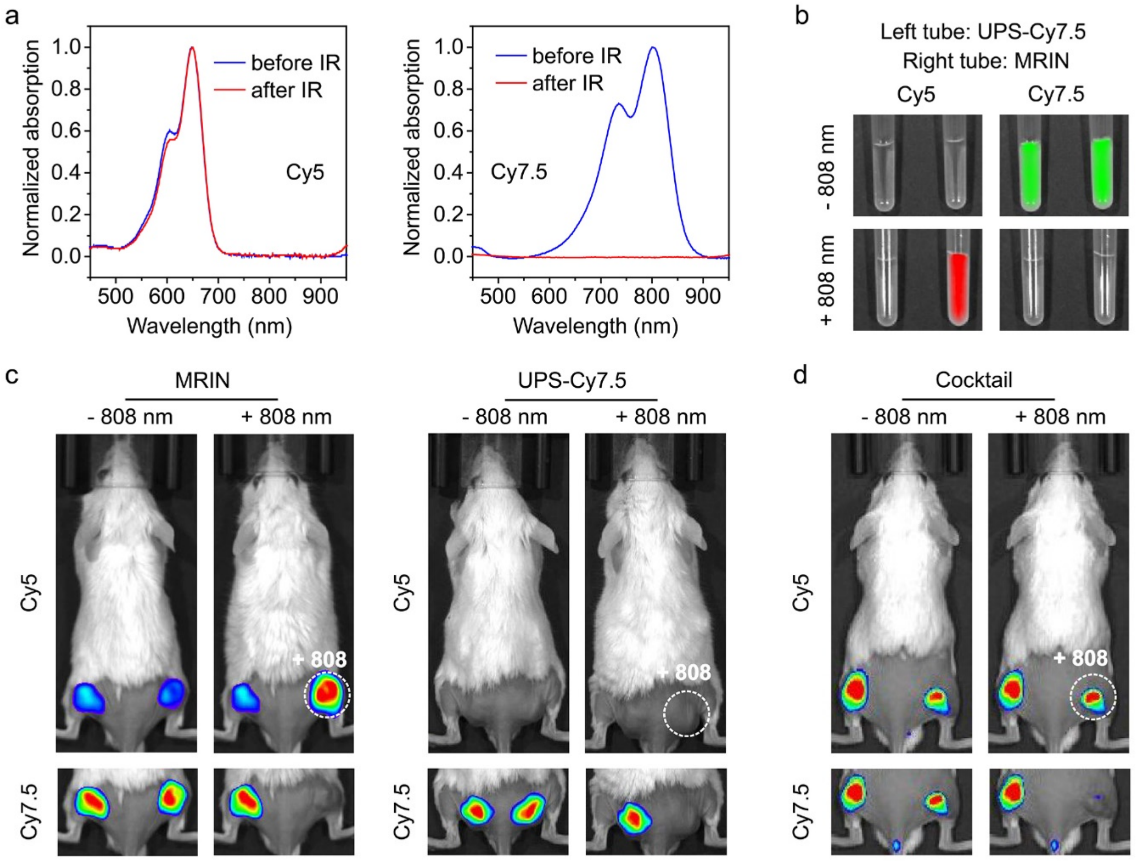

Supplementary Figure 8. The quantitative accuracy of MRIN. (a) UV–Vis spectra of UPS-Cy5 and UPS-Cy7.5 micelles before and after 808 nm laser irradiation, respectively. (b) The fluorescence images of UPS-Cy7.5 micelles and MRIN dispersed in pH 7.4 PBS with or without laser irradiation. (c) The mice bearing bilateral 4T1 tumours were intravenously administrated with UPS-Cy7.5 micelles or MRIN (equivalent Cy7.5 concentration of 0.375 mg kg^{-1}) at 24 h post-injection, the right tumours were irradiated with 808 nm laser for 10 min, the pre- and post-irradiated fluorescence images were captured by IVIS. (The circles indicate the irradiation sites). (d) The mice were injected intravenously with cocktail micelle at 24 h post-injection, the right tumours were irradiated by 808 nm laser for 10 min, and the fluorescence images were collected using IVIS imaging system before and after irradiation. (The circles indicate the irradiation sites).

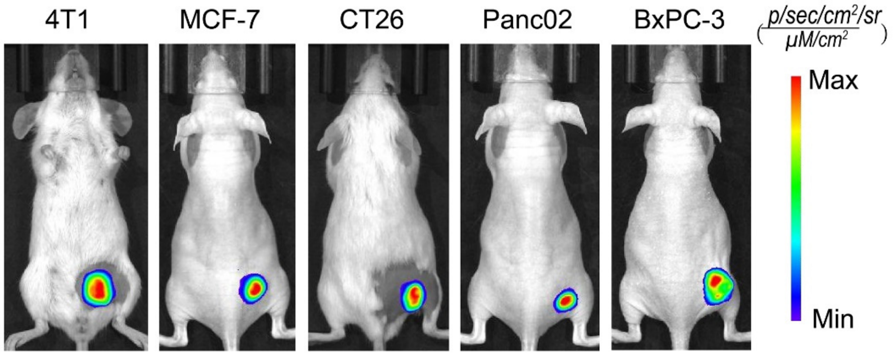

Supplementary Figure 9. The total nanoparticle accumulations in different tumour models (4T1, MCF-7, CT26, Panc02, and BxPC-3) at 24 h post-injection of MRIN.

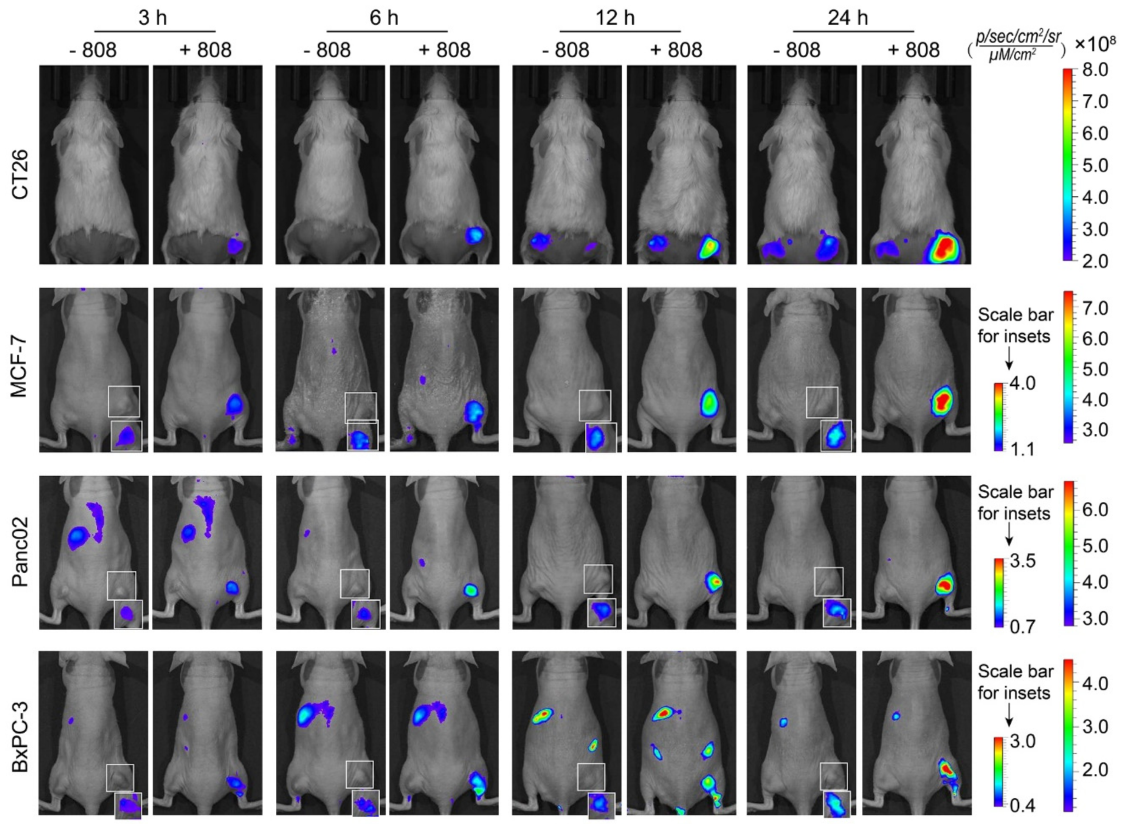

Supplementary Figure 10. *In vivo* Cy5 fluorescence images of the bilateral CT26, MCF-7, Panc02, and BxPC-3 tumour-bearing mice with or without laser irradiation on right tumours at 3, 6, 12 and 24 h post-injection of MRIN. The squares indicate the irradiation sites of right tumours. During irradiation, the real-time temperature of the tumours was kept at about 42 °C. Insets are rescaled images.

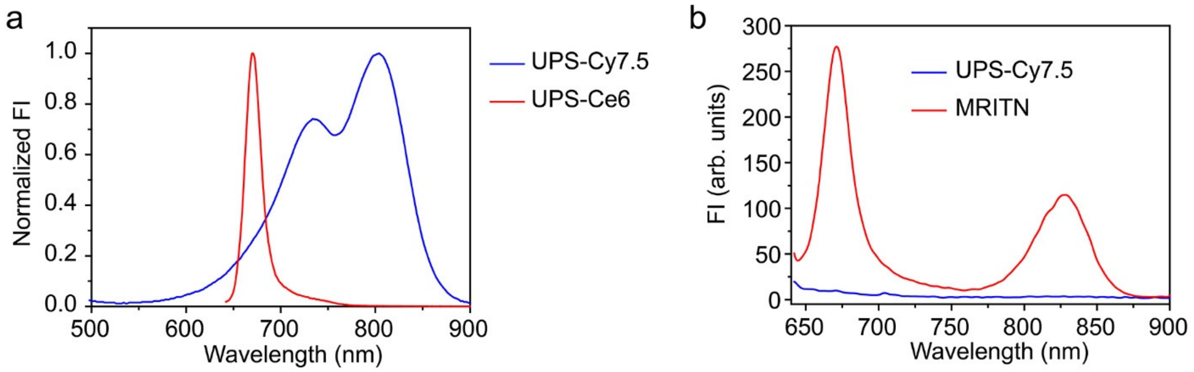

Supplementary Figure 11. The FRET effect between Ce6 and Cy7.5. (a) The emission spectrum of Ce6 and the excitation spectrum of Cy7.5. There is good overlap between them, enabling the FRET effect from Ce6 to Cy7.5. (b) The emission spectra of MRITN (UPS-Ce6/UPS-Cy7.5 hybrid micelle) and UPS-Cy7.5 micelles at 630 nm for Ce6 excitation. UPS-Cy7.5 micelle had no emission peak at 825 nm with excitation at 630 nm, while MRITN has two emission peaks at 670 nm and 825 nm, respectively, indicating that Cy7.5 can absorb the energy of Ce6 emission to emit its own signal.

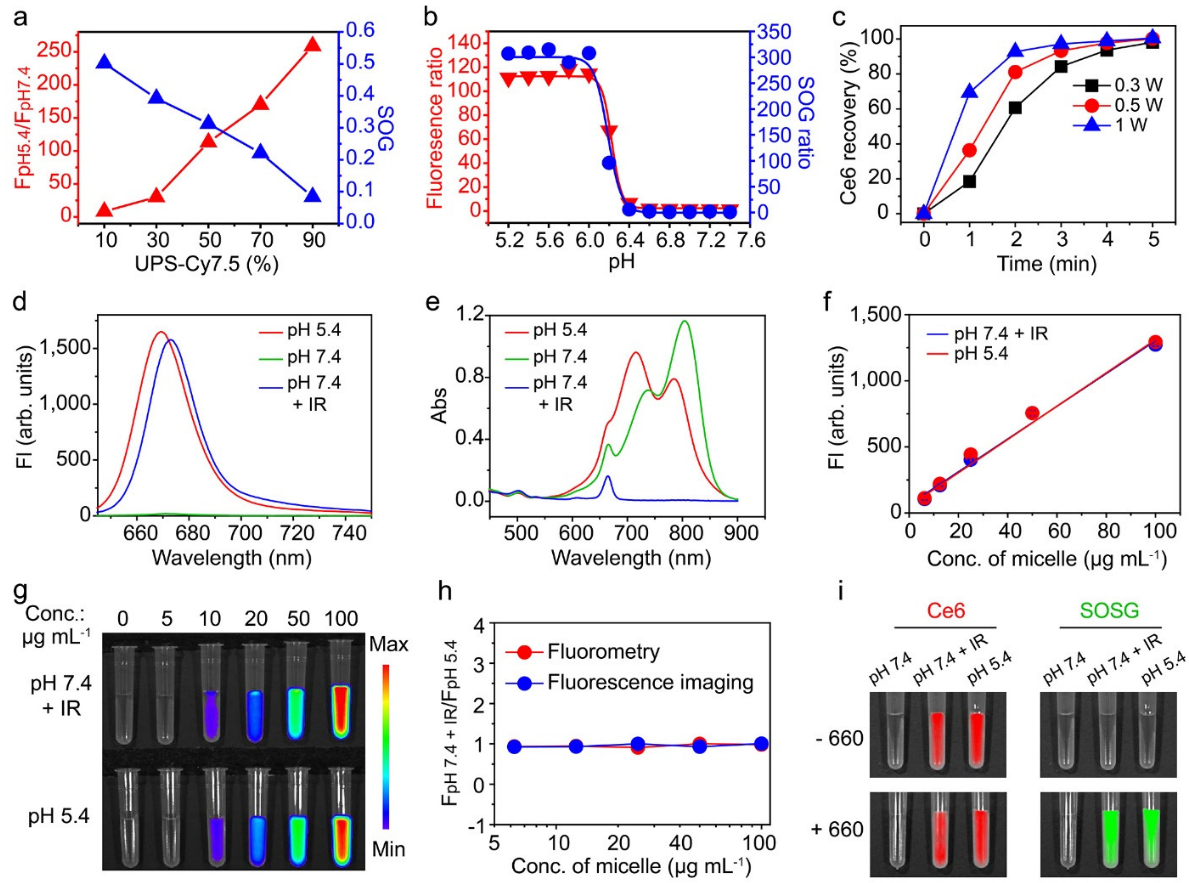

Supplementary Figure 12. Development and characterization of MRITN. (a) Ce6 fluorescence intensity ratio and SOG production as a function of molar ratio of UPS-Cy7.5 to UPS-Ce6 polymers in the hybrid micelles. (b) Ce6 fluorescence and SOG production ratios as a function of pH for MRITN. (c) Light-triggered Ce6 fluorescence recovery of MRITN as a function of time at different laser power. (d-e) The fluorescence spectra (d) and UV–Vis spectra (e) of MRITN at pH 5.4 and pH 7.4 with or without 808 nm irradiation (λex/em= 400/670 nm). (f) Linear relationship of Ce6 emission intensity versus the concentration of micelles recorded by fluorescence spectrophotometer. Data were presented as mean ± s.d.. (*n* = 3 biologically independent experiments). (g) Fluorescence images of micelles dispersed in pH 5.4 or pH 7.4 PBS buffers with 808 nm irradiation. (h) The comparison of Ce6 fluorescence recovery by pH-triggered micelle dissociation and light-induced Cy7.5 photobleaching. Data were presented as mean ± s.d.. (*n* = 3 biologically independent experiments). (i) The UPS-SOSG/Ce6/Cy7.5 micelles were dispersed in pH 5.4 and pH 7.4 PBS buffers with/without 808 nm irradiation, then the fluorescence images of micelle solutions with/without 660 nm irradiation were captured by IVIS system.

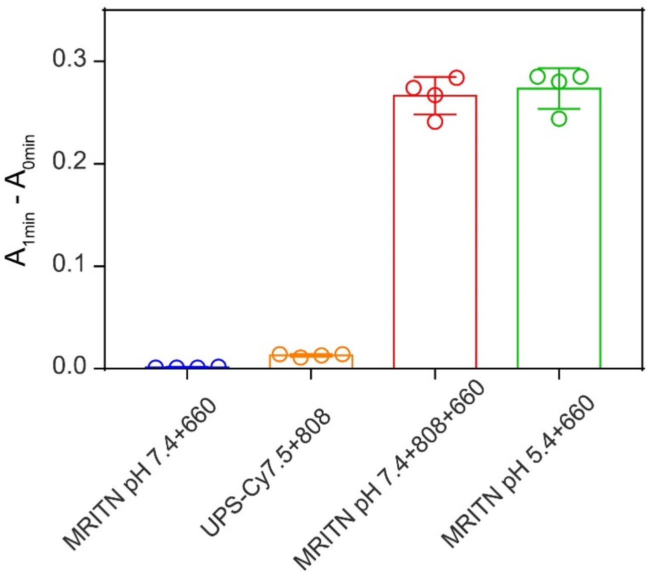

Supplementary Figure 13. The effect of Cy7.5 on photodynamic therapy. The single oxygen generation (SOG) was estimated using N, N-Dimethyl-p-nitrosoaniline (RNO) method. Data were presented as mean ± s.d. (n = 4 biologically independent experiments). At pH 7.4, MRITN is micelle state with Ce6 signal "OFF", therefore, it generates little singlet oxygen under 660 nm irradiation. The Ce6 signals of MRITN can be fully activated through both pH-induced micelles dissociation and 808 nm irradiation-induced Cy7.5 photobleaching, resulting in about 270-fold higher SOG than Ce6 "OFF" state under 660 nm irradiation. However, the singlet oxygen generated by UPS-Cy7.5 with 808 nm irradiation was 20-fold lower than MRITN with 660 nm irradiation in activated Ce6 state. Therefore, the SOG of Cy7.5 was negligible for the photodynamic effect of MRITN.

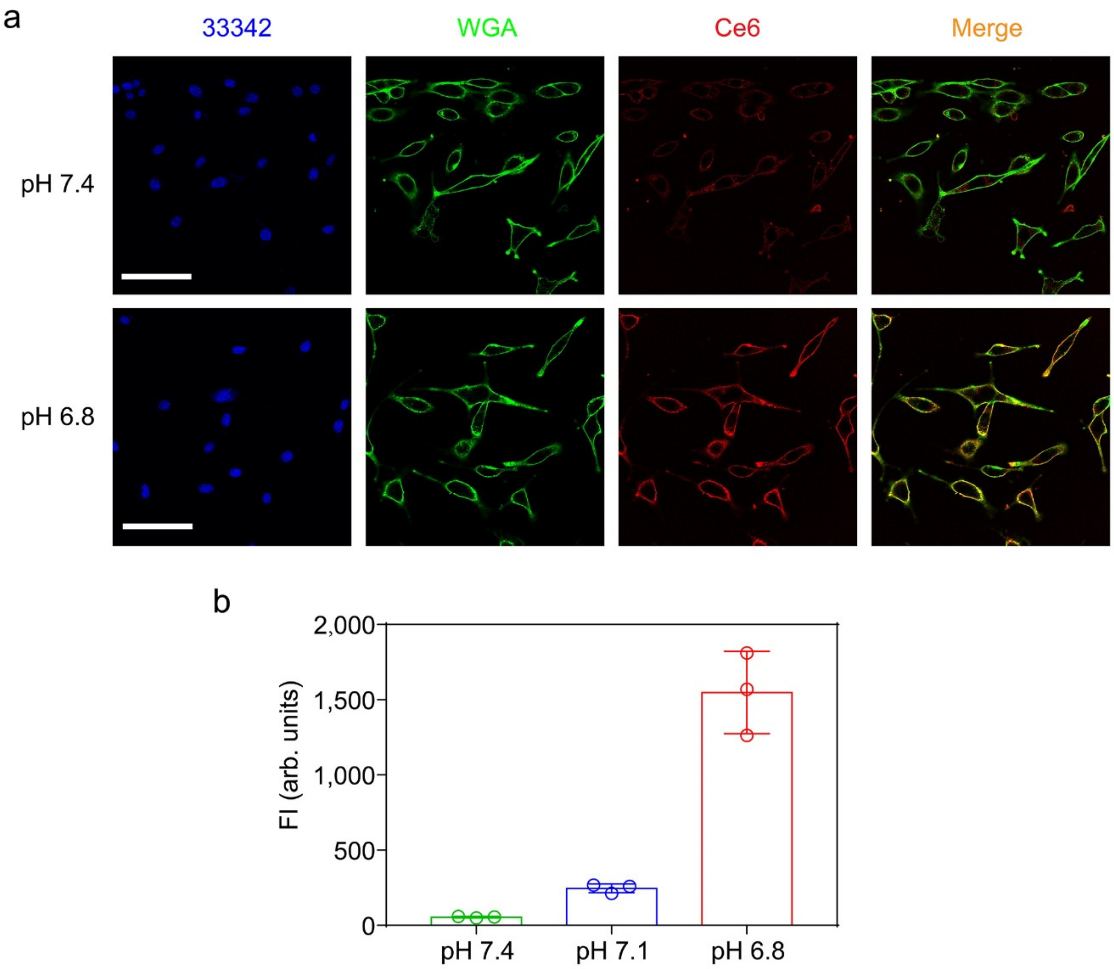

Supplementary Figure 14. The cell membrane binding ability of MRITN at different pH values. (a) 4T1 cells were incubated with MRITN (pre-irradiated with 808 nm laser) in pH 6.8 or pH 7.4 RPMI 1640 medium at 4 °C for 30 min. The fluorescence images were captured by confocal microscope after washing out the extracellular micelles. The cell nuclei were stained with Hoechst 33342 and were shown in blue. The cell membranes were stained with AF488-WGA and were shown in green. The Ce6 signals were presented in red. Scale bar = 100 μm. (b) 4T1 cells were incubated with MRITN (pre-irradiated with 808 nm laser) in pH 6.8, pH 7.1, or pH 7.4 RPMI 1640 medium at 4 °C for 30 min. The cell binding of MRITN based on Ce6 signal was analyzed by flow cytometry. Data were presented as mean ± s.d. (n = 3 biologically independent cell samples).

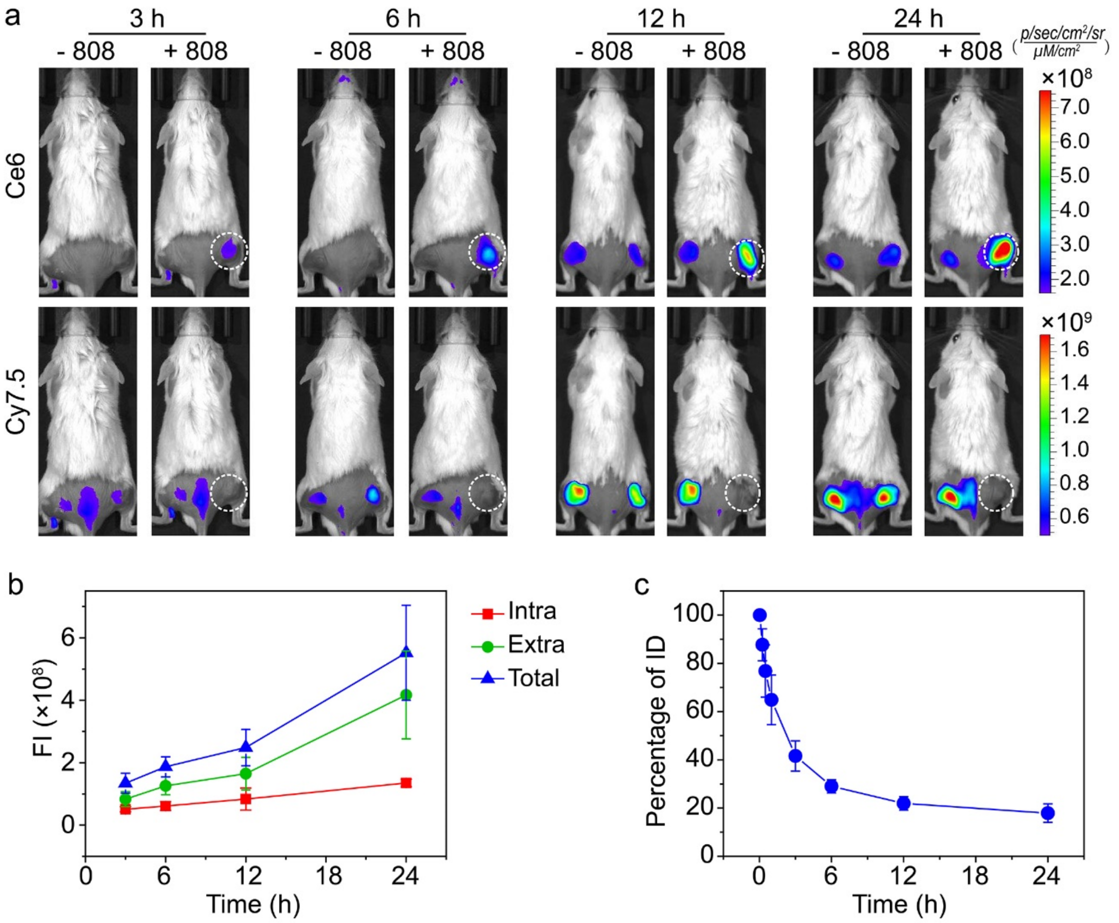

d

$t_{1/2,\ \alpha}$ (h)	$t_{1/2,\ \beta}$ (h)	AUC $_{(0\text{-}t)}$ (mg/ L*h)	AUC $_{(0\text{-}\infty)}$ (mg/ L*h)	MRT $_{(0\text{-}t)}$ (h)
0.59	18.79	717.37	948.86	13.55

Supplementary Figure 15. *In vivo* imaging and pharmacokinetics of MRITN. (a) *In vivo* fluorescence images of the bilateral 4T1 tumour-bearing mice with or without irradiation on right tumours at 3, 6, 12, and 24 h post-injection of MRITN (equivalent Ce6 concentration of 0.75 mg kg^{-1}). The circles indicate the irradiation sites of right tumours. (b) The intracellular, extracellular, and total distributions of nanoparticles in 4T1 tumours at different time post-injection (*n* = 4 biologically independent mice). (c) Plasma concentrations of Ce6 versus time curve for MRITN (*n* = 5 biologically independent mice). (d) The pharmacokinetics parameters of MRITN *in vivo*. All data were presented as mean ± s.d..

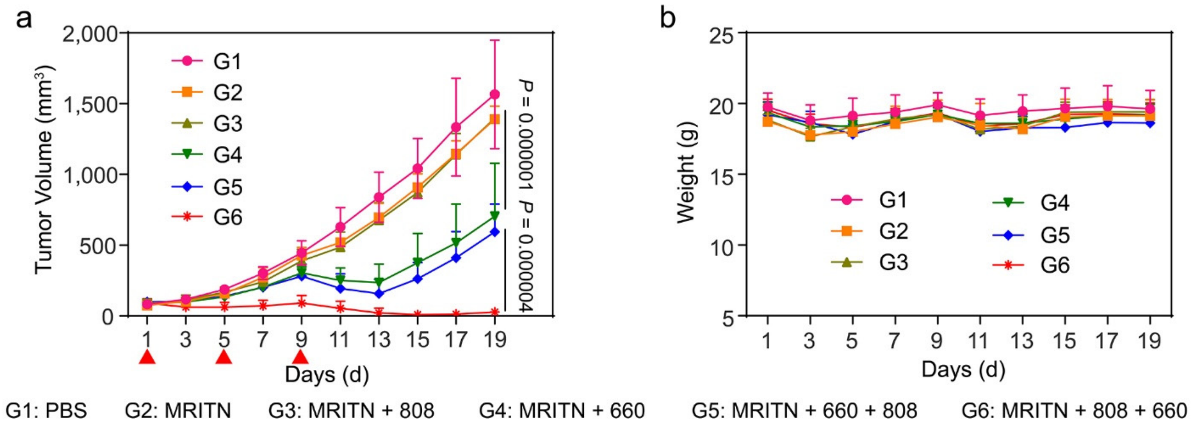

Supplementary Figure 16. The anti-tumour study of MRITN with drug-light interval of 24 h. (a) Tumour growth curves in subcutaneous 4T1 tumour-bearing mice with different irradiation treatments. Mice were irradiated with 660 nm laser at 400 mW cm^{-2} for 10 min at 24 h post-injection of MRITN (Ce6 dose of 0.75 mg kg^{-1}). The PDT treatment was given three times separately at day 1, day 5 and day 9 (n = 6 biologically independent mice). (b) Body weights of mice after initial PDT treatment (n = 6 biologically independent mice). All data are presented as mean ± s.d.. Statistical analysis was performed by two-way analysis of variance (ANOVA).

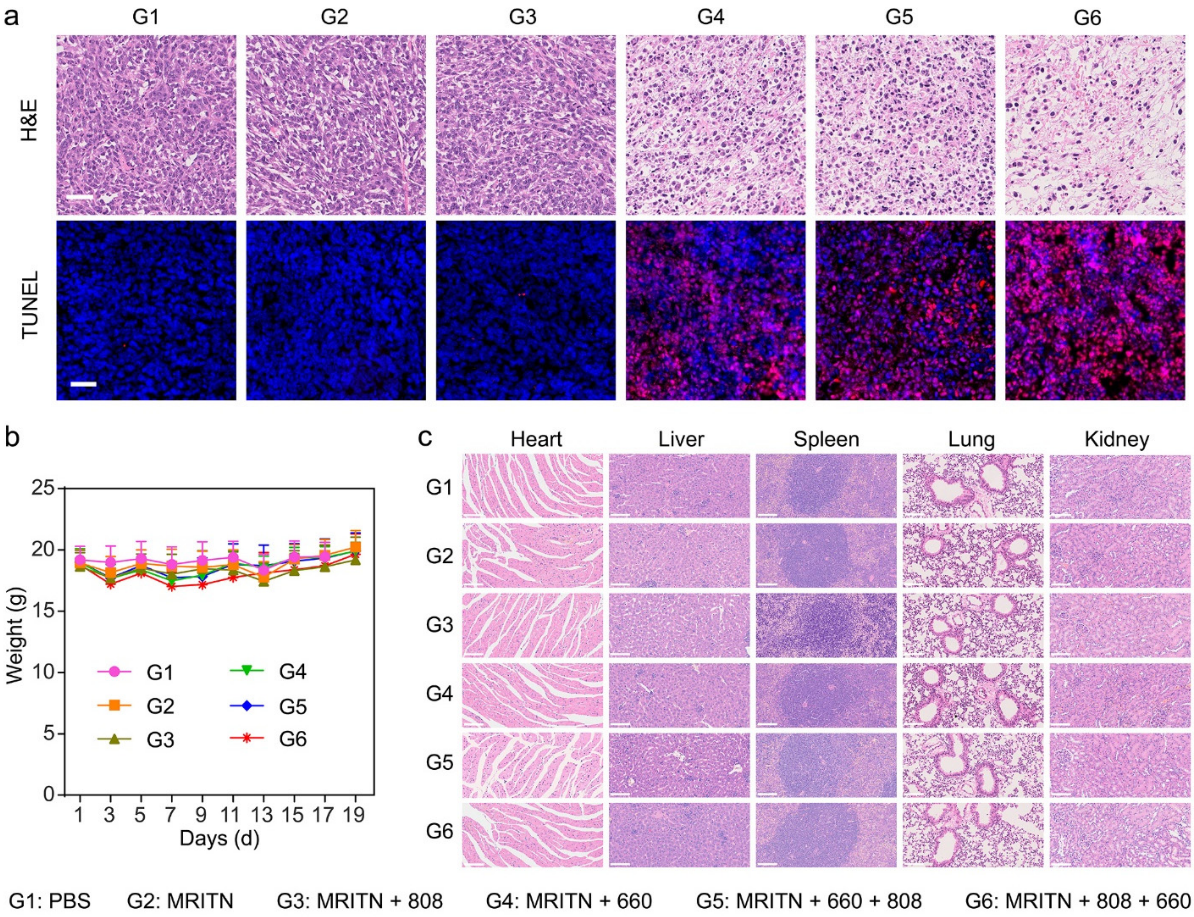

Supplementary Figure 17. (a) Tumour cell apoptosis evaluated by H&E staining and TUNEL analysis, respectively. Scale bar = 50 μm. (b) Body weights of mice after initial PDT treatment. Data were presented as mean ± s.d.. (*n* = 8 biologically independent mice). (c) H&E staining images of the major organ sections collected from different treatment groups of mice at the end of anti-tumour study. Scale bar = 100 μm.

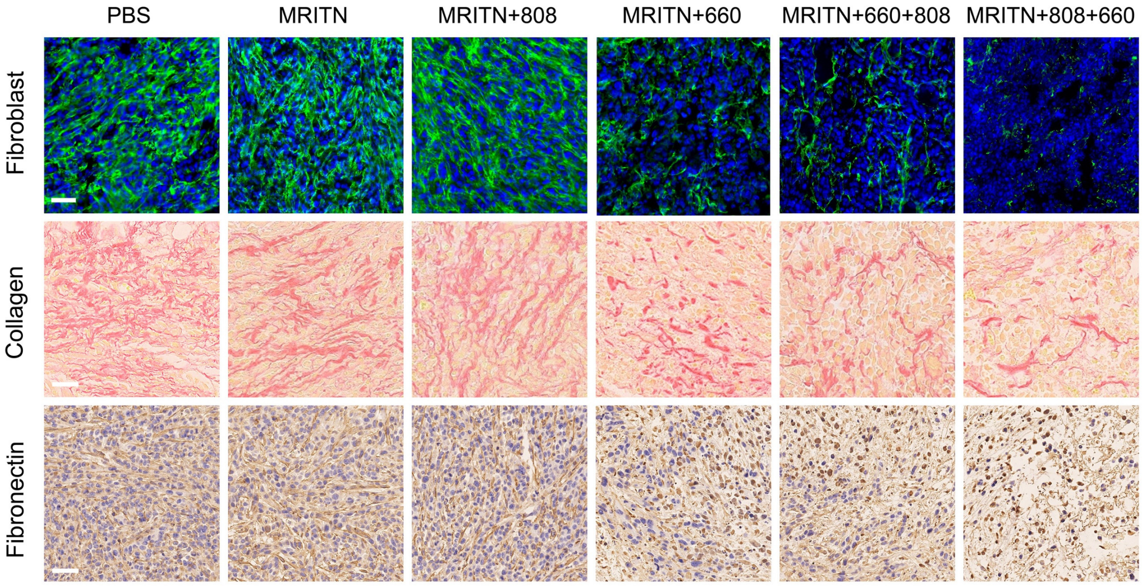

Supplementary Figure 18. Combined PDT remodeled the tumour microenvironment. 4T1 tumours from mice in different groups were excised, sectioned, and stained for tumour associated fibroblasts (CAFs), collagen, and fibronectin, respectively. Scale bar = 50 μm.

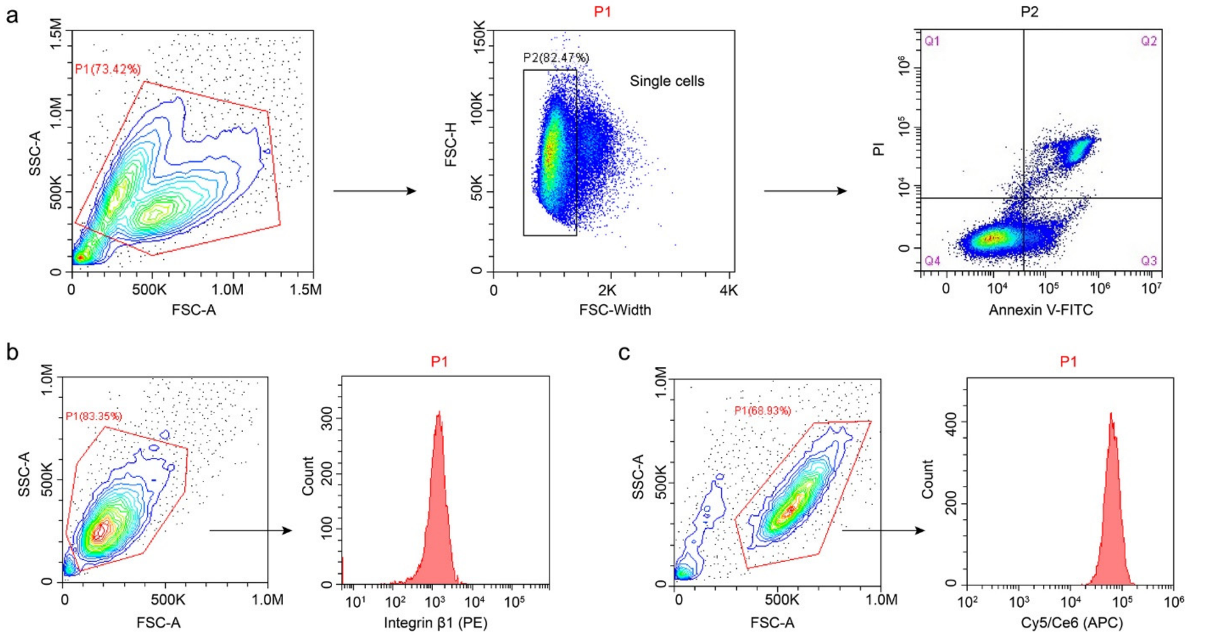

Supplementary Figure 19. FACS gating strategies for (a) apoptosis analysis (related to Figure 5c), (b) integrin β1 expression analysis (related to Figure 6e), and **(c)** cellular uptake and cell membrane binding of NPs (related to Supplementary Fig. 4b and Supplementary Fig. 14b).

Supplementary Table 1. The polymer composition and their ratios for each nanoparticle.

Nanoparticle	Polymer 1	Polymer 2	Polymer 3	Ratio (%)
$RNP_{0\%}$	PEH-$Cy5_1$	PEH-$Cy7.5_3$	Blank PEH	5:45:50
MRIN	UPS-$Cy5_1$	UPS-$Cy7.5_3$	Blank UPS	5:45:50
$RNP_{100\%}$	UPS-$Cy5_1$	/	Blank UPS	5:95
MRITN	UPS-$Ce6_1$	UPS-$Cy7.5_3$	/	50:50

[a] UPS is the abbreviation of ultra-pH-sensitive PEG_{5k}-*b*-P(DPA_{40}-*r*-EPA_{40}-*r*-AMA_3) copolymer.
[b] PEH is the abbreviation of pH-insensitive PEG_{5k}-*b*-P(EH_{80}-*r*-AMA_3) copolymer.
[c] The right subscript of the dye represents dye conjugated numbers.
